# Progress and Impacts of “Omics” Technologies in Understanding the Drought Response in Cassava: Adoption for Food Security in Africa

**DOI:** 10.1002/pei3.70100

**Published:** 2025-12-15

**Authors:** Ambesa Mantewu, Sandiswa Figlan, Amelework Assefa, Molemi Rauwane

**Affiliations:** ^1^ Department of Botany Nelson Mandela University Port Elizabeth South Africa; ^2^ Department of Agriculture and Animal Health, School of Agriculture and Life Sciences, College of Agriculture and Environmental Sciences University of South Africa Roodepoort South Africa; ^3^ Agricultural Research Council‐Vegetable, Industrial and Medicinal Plants (ARC‐VIMP) Pretoria South Africa

**Keywords:** cassava, climate change, drought stress, food security, omics tools

## Abstract

Cassava is a crucial staple crop in Africa, but its productivity is increasingly threatened by the worsening impacts of drought caused by climate change. To address this challenge, an integration of “omics” and genome editing technologies has emerged as indispensable tools for understanding the complex mechanisms that govern cassava's response to drought stress. This article provides an overview of the progress and significant contributions of “omics” technologies, including genomics, transcriptomics, proteomics, and metabolomics, in elucidating the molecular basis of cassava's ability to withstand drought stress. Through the integration of multiple “omics” approaches, researchers have identified key genes, single nucleotide polymorphisms, proteins, and metabolites that are associated with drought stress, offering promising opportunities for the development of drought‐tolerant cassava varieties. Moreover, this review emphasizes the adoption of “omics” technologies to accelerate breeding programs, enhance crop resilience, and ensure food security throughout Africa. By synthesizing current research findings and technological advancements, this review underscores the transformative potential of “omics” technologies in understanding and mitigating the detrimental effects of drought on cassava production, ultimately strengthening food security in Africa.

## 1 Introduction

Cassava, often referred to as a “miracle of the tropics” plays a crucial role in ensuring food security for more than a billion individuals around the world (Lebot 2020). The crop is a starchy root vegetable that is cultivated in tropical and subtropical regions around the world and holds significant nutritional and economic relevance (Amelework et al. 2021). Cassava particularly holds its significance because it serves as a primary source of nourishment worldwide, particularly in Africa (Ceballos et al. 2020), providing essential carbohydrates (McCallum et al. 2017; Putpeerawait et al. 2017) and contributing to food security (Okwuonu et al. 2021). According to Gogoi et al. (2019), cassava ranks as the third most widely consumed plant‐based carbohydrate source, following rice and maize. In terms of its starch content, cassava roots contain approximately 85% starch on a dry‐weight basis (Pan et al. 2021). The high starch yield of cassava has long underscored its potential for industrial applications, particularly in bioethanol production (Zidenga et al. 2012). Cassava has emerged as a prominent feedstock for bioethanol production in various regions, especially in countries like Nigeria, Thailand, and Malaysia (Abotbina et al. 2022; Kongsil et al. 2024). Continuous advancements in pretreatment and fermentation technologies are enhancing both the yield and sustainability of bioethanol production from this crop (Nanssou et al. 2016; Afedzi et al. 2025). Moreover, cassava exhibits potential as an alternative industrial crop because of its versatility, boasting over 300 industrial applications, including the production of tyres, adhesives, medicines, animal feeds, cold meats, and alcohol (Amelework et al. 2021).

This versatile crop possesses unique attributes that allow it to thrive in challenging conditions, where other essential food crops encounter difficulties. Consequently, it serves as an exceptional solution for adapting to the effects of climate change (FAO 2018), particularly in the African tropics, where the environment is characterized by high temperatures and aridity (Chevalier and Chase 2016; Daramola et al. 2023). Originating in South America, cassava was introduced to Africa during the colonial era and has since evolved into a staple food crop for many people on the continent (Iwuagwu 2012). According to FAO (2018), the annual consumption of cassava in Africa in 2018 amounted to 50 kg per capita. It is worth noting that the sub‐Saharan African (SSA) region exhibited the highest per capita cassava consumption, estimated at 800 g per person per day. Consequently, this crop fulfills a significant portion of the population's energy requirements in the SSA region (Nhassico et al. 2008; Burns et al. 2010). In 2019, Africa significantly contributed over 63% of the global production of cassava, which amounted to a staggering 303 million tons (FAO 2018). In SSA, approximately 40 countries are involved in the production of cassava, accounting for 61.1% of the global cassava production (FAOSTAT 2020). Cassava's capacity to thrive in marginal environments positions it as an appreciated crop for ensuring food security in SSA, where it serves as one of the staples. Nonetheless, a notable issue arises from the impact of drought on cassava's yield production, despite its ability to endure such conditions (Daryanto et al. 2016). Consequently, this poses a significant threat to SSA's overall food security.

Over the years, drought continues to be a significant limitation on crop productivity, especially in the context of increasing climate variability (Cotrina Cabello et al. 2023). Although the general mechanisms of plant drought resilience, including avoidance, tolerance, escape, and recovery, are well established (Fang and Xiong 2015), the molecular underpinnings of these mechanisms in cassava have only recently been elucidated through integrative omics approaches. Cassava demonstrates a significant ability to avoid drought through early stomatal regulation and the deposition of cuticular wax (Oliveira et al. 2015). For instance, transcriptomic profiling has associated drought avoidance in cassava with the rapid down‐regulation of genes involved in stomatal development (*GC1*, *OST1*) and the activation of pathways related to cutin and wax biosynthesis in cassava leaves (Orek 2023). However, its remarkable resilience is primarily ascribed to post‐stress recovery and the maintenance of metabolic function during extended periods of drought stress. This characteristic is associated with dynamic transcriptional reprogramming in both the roots and leaves as described by Orek et al. (2020). Omics‐enabled insights indicate that cassava's drought response is a genetically encoded, tissue‐specific, and developmentally regulated process, rather than a generic stress reaction.

Cassava can withstand drought, although at the expense of decreased root production, which is the edible and nutritious part of the plant (Daryanto et al. 2016). Under drought stress, the average productivity of cassava is considerably lower than its potential yield, which indicates room for genetic advancements to close yield gaps (More et al. 2023). With the change in climate, drought is increasingly becoming a more frequent and severe problem for crops like cassava (Pipitpukdee et al. 2020; Pushpalatha and Gangadharan 2020). Understanding the molecular mechanisms behind the crop's drought response is essential to comprehending cassava's response to drought, and resilient cultivars that can thrive under these conditions can be developed. This information will not only assist in improving the crop's yield and production but also contribute to the conservation of natural resources and ensure food security, particularly in low‐income countries (Okogbenin et al. 2013; Turyagyenda et al. 2013; Orek et al. 2020; Pushpalatha and Gangadharan 2020). Thus far, efforts have been made to incorporate advanced omics tools for dissecting complex mechanisms and improving crops exposed to drought conditions (Fu et al. 2016; Mo et al. 2018; Shan et al. 2018; Ding et al. 2019; Wu et al. 2019; Suksamran et al. 2020; Yan et al. 2020; dos Santos Silva et al. 2021; Wang et al. 2021; Zhang et al. 2021; Boutsika et al. 2022; Guo et al. 2022; Ali et al. 2023; Ghorbanzadeh et al. 2023).

Omics technologies provide perspectives and opportunities for understanding the molecular underpinnings of stress response in plants, leading to advancements in enhancing plant abiotic and biotic stress tolerance (Duque et al. 2013; Li et al. 2021; Ponce et al. 2022; Liang et al. 2023). Using omics technologies enables the possibility of identifying regulatory networks in complex hubs and evaluating multiple targets simultaneously. These technologies enable the identification and characterization of molecular components such as transcripts, single nucleotide polymorphisms (SNPs), proteins, and metabolites, aiming to reveal functional networks and models that can predict an organism's behavior in response to various environmental conditions like drought (Pandian et al. 2020). They have also been useful in understanding drought stress responses in several root crops such as carrots, potatoes, and sweet potatoes (Sprenger et al. 2018; Boutsika et al. 2022; Öztürk Gökçe et al. 2022; Zhou et al. 2022), including cassava (Utsumi et al. 2012; Wang, Yu, et al. 2014; Hu et al. 2015; Li et al. 2017; Shan et al. 2018; Ruan et al. 2018; Ding et al. 2019; Ewa et al. 2021; Wang et al. 2021). Moreover, omics technologies are becoming game changers for food crops in Africa (Benkeblia 2014; Nayak et al. 2021), where agriculture is hampered by problems such as poor infrastructure, pests, diseases, and abiotic stresses, such as drought (Gbashi et al. 2021; Kavhiza et al. 2022). With the knowledge gained from the results of omics technologies, breeders can develop high‐yielding and stress‐tolerant cultivars suitable for the African agroecological conditions (Gedil et al. 2016; Yang et al. 2021; Derbyshire et al. 2022), thus addressing the continent's challenges with food and nutrition security (Mmbando 2024). Overall, integration of omics technologies in African crop breeding programs proved to be a powerful and instrumental tool at our disposal to achieve multiple Sustainable Development Goals (SDGs), including the fundamental eradication of hunger under the ever‐changing environmental conditions. This review showcases the exciting strides made in the field of molecular biology through the utilization of “omics” approaches in improving cassava's ability to withstand drought conditions. Furthermore, this review will examine the accomplishments as well as challenges associated with using “omics” technologies to enhance cassava resilience to drought stress in Africa.

## 2 “Omics” Approaches

Omics technologies encompass a comprehensive examination of the biological molecules or components that constitute an organism (Karahalil 2016). The utilization of omics technologies, including genomics, transcriptomics, proteomics, and metabolomics, facilitates an understanding of the genetic, molecular, biochemical, and physiological mechanisms contributing to the plants' ability to respond to and withstand environmental factors (Mehta, James, et al. 2019). This understanding enables scientists to design strategies that enhance the crops' tolerance to stress, in which particular emphasis has been given to the increasing frequency and severity of environmental conditions worldwide (Schneider and Orchard 2011). Extensive research has been conducted on the application of omics technologies to understand stress responses in Africa's staple crops. For instance, genomic studies on crucial crops like maize, wheat, cassava, and rice have facilitated the identification of genes associated with important traits such as yield, pest and disease resistance, and nutritional value, among others (Damaris et al. 2016; Gowda et al. 2015; Guo et al. 2018; Karmakar et al. 2019; Nyaga et al. 2019; Scarcelli et al. 2019). Proteomics and metabolomic studies have provided insights into the metabolic pathways and expression patterns among crops under various environmental conditions, including drought (Benevenuto et al. 2017; Shan et al. 2018; Chang et al. 2019; Yajie et al. 2021; Ali et al. 2023). Through genomics approaches, understanding the response of plants against environmental conditions was achieved, which led to the development of cultivars with disease resistance (Wolfe et al. 2016; Brito et al. 2017; Ozimati et al. 2022; Ntui et al. 2024), enhanced drought tolerance (Yu et al. 2021; Singh et al. 2022), and improved nutritional values, among others (Rana et al. 2020).

Omics technologies for enhancing African food crops are currently attracting growing interest and witnessing positive advancements (Ibraheem et al. 2018; Ribaut and Ragot 2019; Kumar et al. 2021). Although the acceptance and utilization of omics technologies in African agriculture are still evolving, African agricultural institutions and international partnerships are increasingly prioritizing these approaches. Several initiatives and research projects have commenced investigating the proteomics, genomics, and metabolomics of African crops (Ghazal et al. 2021; Mmbando 2024). These studies aim to elucidate the genetic foundations of important traits, comprehend crop responses to environmental stresses, and identify molecular markers for crop improvement. Substantial efforts have also been made to establish genomic resources for key food crops in Africa. The African Orphan Crops Consortium (AOCC) is one of the international research initiatives established to improve the nutrition, productivity, and climatic adaptability of some of Africa's most important food crops. The AOCC aimed to sequence, assemble, and annotate the genomes of 100 traditional African food crops (Hendre et al. 2019). Genome sequencing projects have resulted in the availability of reference genomes for various crops, including maize (Chandler and Brendel 2002), rice (Wang, Feng, et al. 2014), wheat (IWGSC 2014), sorghum (Mace et al. 2013), and cassava (Prochnik et al. 2012). These resources serve as fundamental tools for understanding crop genetics, locating potential SNPs and genes, and enhancing breeding strategies. Omics technologies have also facilitated the identification of genes, SNPs, proteins and metabolites associated with important agricultural traits in African crops (Gedil and Menkir 2019; Faryad et al. 2021; Yang et al. 2021). By adopting the use of omics technologies, the findings have the potential to address agricultural challenges facing Africa and offer promising opportunities for crop improvement (Mmbando 2024).

The following sections will provide a review of the progress made by omics technologies, including genomics, transcriptomics, proteomics, and metabolomics, in uncovering the intricate pathways underlying cassava responses to drought stress in an African context.

### 2.1 Genomics

The first draft reference genome of cassava was released in November 2009 (Prochnik et al. 2012), and once released, certain features of the genome were highlighted or taken advantage of to address challenges with growth and sustainability of the crop, particularly in Africa. The sequence and annotation of the cassava genome (Prochnik et al. 2012; Bredeson et al. 2016) have helped shed some light on the mechanisms behind the crops' response to environmental stress, including drought (Nuñez‐Muñoz et al. 2021), which is a major constraint to cassava production throughout Africa. The enhanced accessibility of genomic resources has facilitated the analysis of genotypic data and its application in gaining a deeper comprehension of genotypic variation in African cassava germplasms. These outcomes were derived from next‐generation DNA sequencing technologies, such as genotyping by sequencing, which enable the utilization of Genome‐Wide Association Studies (GWAS) to examine the genetic diversity of cassava germplasm more efficiently (Santos et al. 2022). GWAS allow for a more thorough exploration of important traits' variations, which could help in addressing the challenges presented by polygenic traits in crops like cassava. Considering this, by using genome‐wide marker data, genomic areas linked to drought stress may be more effectively analyzed using GWAS as described by More et al. (2023).

Using GWAS tools to find novel genes imparting drought tolerance is one of the practical methods for identifying new or desired genes in cassava (Table 1). For example, a GWAS study was carried out to investigate the responses of cassava to cold and drought stresses which led to the first reference catalog of 682 high‐confidence Long Non‐Coding RNAs (*lncRNA*s) (Li et al. 2017). Three hundred and eighteen (318) *lncRNA*s that were sensitive to drought and/or cold stresses were found to be frequently co‐expressed in unison or discordance with neighboring genes. The *lncRNA*s were reported to be involved in the generation of secondary metabolites, the transmission of hormone signals, and the metabolism of sucrose, according to an analysis of their trans‐regulatory network (Li et al. 2017). Another GWAS study was carried out, which led to the identification of 56,840 putative non‐coding RNAs that may be involved in the post‐transcriptional regulation of stress‐induced transcription factors (Suksamran et al. 2020). Of these, 3.92% of the *Me‐lncRNA*s have been recognized as potentially novel *lncRNA* transcripts, of which 47 and 51 were down‐ and up‐regulated in response to drought conditions, respectively. Moreover, dos Santos Silva et al. (2021) identified 62 SNPs across 18 cassava chromosomes, which comprised 160 transcripts, including those related to proteins involved in drought tolerance, many of which are conserved in West and East African germplasm. Notably, in Nigeria, the Kompetitive Allele Specific PCR for Single Nucleotide Polymorphism (KASPar SNP) genetic map was developed to serve as a tool to examine traits related to the productivity of cassava plants subjected to moderate drought stress and diseases (table 1; Ewa et al. 2021). The study employed 505 polymorphic SNP markers strategically placed across 21 linkage groups, and 27 Quantitative Trait Locus (QTLs) were identified, specifically linked to 11 productivity traits. Notable findings were reported, including the identification of QTLs *c3loc84.0*, *c6loc0.0*, and *c7loc13.0*, which demonstrated associations with consistent productivity under moderate drought stress conditions. This information is useful in both association mapping and conventional mapping projects, whether generated globally or within Africa, as it accelerates the discovery rate of tolerance genes for drought and other stresses of cassava. An overview and additional cassava genomic studies are given in Table 1.

**TABLE 1:** Genomic approaches used to understand cassava response to drought stress.

Genomic approach	Findings	Description of findings	Implications for breeding	References
GWAS	682 *lncRNA*s with high confidence were identified.	The *lncRNA*s were linked to the sucrose metabolic route, secondary metabolite production, and hormone signal transduction	Potential markers for selection: These *lncRNA*s can be developed into molecular markers to select for lines with superior metabolic and hormonal responses to drought	Li et al. (2017)
GWAS	Differential expression of the aquaporin (*AQP*) gene family in cassava under drought stress	Up‐regulation of *MePIP2‐1* and *MePIP2‐10* enhances drought tolerance	Direct gene targets for transformation or marker‐assisted selection (MAS): Breeding programs can prioritize selecting for alleles of *MePIP2‐1* and *MePIP2‐10* to improve water‐use efficiency	Putpeerawait et al. (2017)
GWAS	ABRE‐binding factor (*ABF*s) gene regulates *MeBAHs* genes to modulate glycine‐betaine (GB) accumulation in cassava leaves under drought stress	*ABF*s bind to *ABRE* and regulate target gene expression	Key regulatory pathway for engineering osmoprotection: The *ABF‐MeBAH*s pathway is a prime target for genetic engineering or for selecting parents with a strong glycine betaine accumulation trait	Feng et al. (2019)
GWAS	A total of 56,840 putative *lncRNA*s were found, of which 2229 were possible new *lncRNA* transcripts, and 250 had distinct expression patterns in cold or drought stress	*lncRNA*s may have a role in the post‐transcriptional control of stress‐induced transcription factors (TFs), zinc‐finger, and *WRKY* gene families	Unlocks a new layer of genetic control for breeding: Provides a novel set of regulatory markers that could be used to fine‐tune the expression of major drought‐tolerant transcription factors	Suksamran et al. (2020)
GWAS	62 SNPs associated with drought‐responsive‐tolerance proteins in cassava	Associated with drought‐responsive‐tolerance protein synthesis, including *APETALA 2* domain (*AP2*), photosystem II oxygen‐evolving enhancer protein, PR5‐like receptor kinase‐related, beta‐fructofuranosidase/saccharase, leucine zipper, and basic leucine zipper (*bZIP*) transcription factors	Toolkit for genomic selection: These SNPs are directly applicable in genomic selection models to predict and select for superior drought tolerance in breeding populations	dos Santos Silva et al. (2021)
KASPar SNP	505 polymorphic SNP markers across 21 linkage groups	27 QTL identified 11 productivity traits in cassava under moderate drought stress	Enables marker‐assisted breeding for yield under stress: These markers allow breeders to directly select for genomic regions that maintain productivity during drought, a key farmer‐preferred trait	Ewa et al. (2021)
GWAS	29 *ALDH* genes were identified, with *MeALDH7B2*, *MeALDH10A9*, and *MeALDH22A1* being strongly up‐regulated under drought conditions	*ALDHs* play a crucial role in mitigating oxidative stress through the detoxification of aldehydes, thereby enhancing drought resilience	Targets for enhancing oxidative stress tolerance: Selecting for or engineering high expression of these specific *ALDH* genes could lead to cultivars with better recovery and survival after severe drought	Tran et al. (2025)

### 2.2 Transcriptomics

Transcriptome profiling has emerged as a widely applicable and very efficient method for examining gene expressions in response to a wide variety of stimuli (Chen et al. 2010; Zhu et al. 2013; Xie et al. 2015; Zheng et al. 2015; Muthusamy et al. 2016; Zhao et al. 2016). The method has been proven to play a role in understanding how plants react to different abiotic stresses, including drought, using different molecular tools. Initially, microarrays have been the most widely adopted protocol for transcriptome profiling; however, this tool only measures expression levels of genes for which a probe is available (Rensink and Buell 2005). In cassava, early transcriptomic studies under drought stress used microarray technologies. For example, a study by Utsumi et al. (2012) employed Agilent microarray technology, specifically the 60‐mer oligonucleotide microarray, to conduct transcriptomic analysis of various cassava genotypes under drought stress. The findings unveiled around 1300 up‐regulated genes in cassava when exposed to drought stress, suggesting comparable response and tolerance mechanisms to drought in the treated genotypes. In addition, Fu et al. (2016) used a microarray approach to investigate drought stress induced in cassava through the addition of Polyethylene Glycol. The study revealed substantial alterations in gene expression patterns, with the identified genes being implicated in various stress response‐related biological processes. Consequently, valuable knowledge was gained about the molecular mechanisms underlying cassava's reaction to drought stress. In a separate investigation, Ruan et al. (2018) examined the response of two distinct cassava cultivars (Argentina 7 and South China 124) to drought stress. Using the Affymetrix Arabidopsis ATH1 Genome Array, a microarray trial was executed to scrutinize drought‐responsive *CC*‐type glutaredoxin (*GRX*) genes in cassava. The findings confirmed the induction of six *CC*‐type *GRX* genes (*MeGRXC3*, *C4*, *C7*, *C14*, *C15*, and *C18*) in the leaves of both cultivars. It is worth mentioning that the overexpression of the *MeGRXC15* gene influenced the expression of stress response transcription factors. Protein interaction analysis shed light on interactions between *MeGRXC15* and *TGA5* from Arabidopsis, as well as *MeTGA074* from cassava. Overall, this study provided indispensable insights into the role of *CC*‐type *GRX*s in cassava's response to drought and abscisic acid (ABA) treatments.

Apart from probe‐utilizing microarrays, other technologies that do not use probes such as Illumina/Solexa, ABI/SOLiD, 454/Roche/and nanopore (Minion/PaBio) sequencing have been introduced for gene expression profiling purposes (Mardis 2008; Zhang et al. 2011; Greninger et al. 2015; Rhoads and Au 2015; Lu et al. 2016). The availability of these technologies on the market has fast‐tracked the capability to measure the expression of thousands of genes in plant species (Yang et al. 2012; Yan et al. 2020; Wang et al. 2021) in response to both biotic and abiotic stresses. This has opened new opportunities for scientists to identify mechanisms that harness the performance of the traits of interest. For instance, Wang, Yu, et al. (2014); Wang, Feng, et al. (2014) discovered 1678 gene models linked to environmental stresses, including drought in cassava cultivars W14 and KU50, using the Illumina HiSeq2000 and Roche/454 GS FLX platforms. These genes were found to play a role in response to abiotic stresses and control vital processes such as photosynthesis and the structure of photosynthetic organelles that are crucial for the proper functioning of components of cells like plastid organelles and cytoplasts. Furthermore, the genes were also found to be essential for reacting to stimuli like ABA, oxidative stress, and temperature changes. In addition, an Illumina NovaSeq platform was used to evaluate the leaf mesophyll and leaf vasculature of the cassava cultivar KU50 after exposure to mild drought treatment through comparative transcriptome profiling (table 2; Wang et al. 2021). Genes related to photosynthesis, carbohydrate metabolism, and hormone signaling were found to be up‐regulated in the leaf mesophyll, whereas genes related to cell wall modification and stress response were up‐regulated in the leaf vasculature tissue. In a study by Hu et al. (2015), the activation of stress response genes such as *MeCIPK11*, *MeCIPK17*, *MeCIPK19*, and *MeCIPK25* in a SC124 cultivar was reported in response to drought stress using Illumina approaches.

To date, genes associated with drought stress response or resistance in cassava have been reported through the use of the aforementioned technologies. Although certain studies referenced herein exemplify pioneering applications of microarray technology (Utsumi et al. 2012) and RNA sequencing (Wang, Yu, et al. 2014; Wang, Feng, et al. 2014) in the context of cassava drought research, others represent subsequent research that has been conducted to expand upon these foundational efforts. Although many of the mentioned foundational studies used non‐African germplasm in this section, the stress‐response pathways they identified are largely conserved across cassava diversity. This conservation allows African researchers to use global omics resources to prioritize candidate genes, develop molecular markers, and validate trait associations in locally adapted germplasm within field conditions relevant to smallholder farming systems. Collectively, these studies have identified a diversity of genes that play significant roles in cassava crop responses to drought stresses, as summarized in Table 2.

**TABLE 2:** Transcriptomic approaches used to understand cassava response to drought stress.

Transcriptomic approach	Findings	Description of findings	Implications for Breeding	References
Microarray	A set of 1300 genes with increased expression in response to drought‐induced stress were identified	These genes were essentially important for biological processes, molecular functions, and cellular components The genes were either up‐regulated or down‐regulated These are putative genes for high starch content and recovering from stress	Provides a foundational gene set for screening: This early list can be used to identify potential markers for selecting cultivars that maintain starch production under drought	Utsumi et al. (2012)
RNA‐seq	1678 gene models were identified A high level of genetic diversity and many single nucleotide variations Genes with environmental stressors (drought) have been favored through natural selection and domestication	In cultivated varieties, the genes were involved in photosynthesis and shaping photosynthetic organelles, responding to abiotic stressors, cell parts like cytoplast and plasmid organelle, response to a stimulus, for example, ABA, oxidative stress, temperature In wild varieties, the genes were involved in transporter activity, including potassium symporter and calcium transporting ATPase, cell wall polysaccharide biosynthesis process, secondary metabolic process, and response to a stimulus such as water stress	Highlights the value of wild relatives: Suggests that crossing cultivated cassava with wild relatives could reintroduce valuable drought‐adaptive traits lost during domestication, such as efficient ion transport	Wang, Yu, et al. (2014)
RNA‐seq	25 *CIPK* genes were identified The wild subspecies and two cultivated cultivars showed that most *MeCIPKs* had different expression patterns in different tissues or responses to drought	9 *MeCIPK* genes were reported to be responsible for drought stress, osmotic, salt, and cold stimuli, oxidative stressors, and ABA signaling	Identifies key signaling nodes for broad stress tolerance: These *MeCIPK* genes are prime targets for developing markers to select for cultivars with resilience to multiple abiotic stresses	Hu et al. (2015)
Microarray	The regulatory networks that underlie drought stress that depend on and without ABA were identified	Significantly increased genes, including those involved in glycolysis, ethylene and ABA production, lipid metabolism, protein breakdown, and flavonoid secondary metabolism	Maps complex regulatory networks for precise breeding: Enables the selection of parents that possess a balanced activation of both ABA‐dependent and independent pathways for a robust drought response	Fu et al. (2016)
RNA‐seq	1666 differentially expressed genes (DEGs) were reported	The DEGs are related to the synthesis of proteins, the light reaction of photosynthesis, the metabolism of ethylene, and the cell wall	Reveals key processes for maintaining growth under stress: Breeders can focus on selecting for genotypes that minimize the down‐regulation of photosynthesis and protein synthesis during drought	Zeng et al. (2017)
RNA‐seq	Differentially expressed *CIPK* genes were reported in cassava under drought induced by polyethylene glycol	4 *MeCIPK* genes were reported and have responsibilities across several abiotic stressors, including drought	Confirms core *CIPK* genes for MAS: Provides a shortlist of specific, highly relevant *MeCIPK* genes for marker‐assisted selection programs aimed at drought tolerance	Mo et al. (2018)
Microarray	18 different types of *CC‐GRX*s were discovered and activated under drought conditions Six of these *CC‐GRX*s were found to be stimulated by the application of ABA	ABA‐mediated drought signaling	Identifies candidates for improving oxidative stress tolerance: These *CC‐GRX*s can be used as markers to select for lines with enhanced ability to detoxify reactive oxygen species during drought	Ruan et al. (2018)
RNA‐seq	1242 and 715 differentially expressed genes in cassava leaves and roots under drought conditions	DEGs were exclusively regulated at the mRNA level The genes were related to flavonoid biosynthesis, phenylpropanoid biosynthesis, and hormone biosynthesis, highlighting the involvement of post‐transcriptional regulation in the drought response of cassava	Uncovers tissue‐specific defense mechanisms: Supports the development of markers for root‐specific traits (e.g., flavonoid production) that are crucial for soil water exploration and retention	Ding et al. (2019)
RNA‐seq	91 specific genes (*MePOD*s) were discovered	The *MePOD* genes were involved in hormone responses, postharvest physiology, senescence, and responses to biotic and abiotic stress in cassava	Potential for dual‐stress resistance: These genes could be leveraged to breed cultivars with combined tolerance to drought and postharvest deterioration, reducing losses	Wu et al. (2019)
RT‐qPCR	Expression analysis of drought‐responsive genes (DRGs) categorized into ABA‐dependent (ABA‐D) and ABA‐independent (ABA‐I) pathways Genes such as *NCED3*, *RD29A/B*, *SLAC1*, and *SNAC1* were up‐regulated in drought‐tolerant genotypes but down‐regulated in drought‐susceptible ones at early drought stages *OST1* and *DSTP* were up‐regulated in susceptible genotypes, whereas *ABI1*, *PLDα1*, and *MYB44* were up‐regulated after re‐watering in tolerant genotypes *DREB1A/B*, *DREB2A/B*, *RD29A/B*, and *ERD10* were induced under drought in both genotype groups, indicating ABA‐independent responses	Drought tolerance in cassava was associated with sustained stomatal conductance and coordinated regulation of ABA‐mediated and ABA‐independent stress signaling genes Tolerant genotypes maintained higher photosynthetic activity under drought stress through delayed stomatal closure and efficient gene regulation The identified genes serve as potential molecular markers for selecting drought‐resilient cassava lines	Provides a direct molecular toolkit for phenotyping: This set of genes (*NCED3*, *SNAC1*, etc.) can be used in qPCR assays to rapidly screen and identify drought‐tolerant breeding lines in the seedling stages	Orek et al. (2020)
Microarray	The interaction of *MeCIPK* with *MeWHY* genes resulted in the up‐regulation of MeCIPK23 was reported	*MeWHY*s and *MeCIPK23* interact to cause the synthesis of abscisic acid, which enhances cassava's tolerance to drought stress	Reveals a specific regulatory module for engineering: The *MeWHY‐MeCIPK23* interaction is a precise target for genetic engineering to enhance ABA biosynthesis and drought tolerance	Yan et al. (2020)
RNA‐seq	108 DEGs were involved in the mid‐vein vasculature, whereas 116 DEGs were involved in the leaf mesophyll	The DEGs were involved in the biosynthesis and metabolism of amino acids, glutamic acid, starch, and sucrose	Highlights source‐sink relationships under stress: Guides breeding for varieties that efficiently manage carbon and nitrogen partitioning between leaves and storage roots during drought	Wang et al. (2021)
RNA‐seq	A total of 454 DEGs were identified in the drought‐tolerant clone 8S501, whereas 1249 DEGs were identified in the less drought‐tolerant clone H97 under drought conditions There were 322 genes found to be differentially expressed between clones 8S501 and H97 during drought, with 165 genes exhibiting up‐regulation and 157 genes exhibiting down‐regulation in 8S501 Notable up‐regulated genes in clone 8S501 include acyl‐CoA oxidase, glutathione peroxidase, vesicle transport‐associated genes, late embryogenesis abundant (*LEA*) proteins, brassinosteroid (BR)‐signaling kinase, histone demethylase, and major facilitator superfamily (*MFS*) transporter genes	Comparative transcriptomic analysis of tuber tissues has identified novel drought‐responsive genes Aquaporins, transporter genes, calcium binding proteins, and ABA‐related genes were found to be up‐regulated in both clones The drought‐tolerant clone 8S501 exhibited enhanced expression of genes associated with redox regulation, vesicle transport, epigenetic regulation, and carbohydrate metabolism This study underscores the tuber‐specific molecular mechanisms underlying drought tolerance in cassava	Identifies key mechanisms for tuber survival and yield: Provides specific markers (e.g., for *LEA* proteins, redox control) to select for cultivars that protect the economically critical tuber organ under drought	Koundinya et al. (2024)
RNA‐seq	A total of 29 *ALDH* genes were identified within the cassava genome and classified into nine distinct families: *ALDH2*, *ALDH3*, *ALDH5*, *ALDH6*, *ALDH7*, *ALDH10*, *ALDH11*, *ALDH18*, and *ALDH22* 9 specific *ALDH* genes, including *MeALDH2B1*, *MeALDH3I1*, and *MeALDH7B4*, exhibited significant up‐regulation in response to drought stress Notably, *MeALDH7B4* demonstrated the highest fold‐change among the evaluated genes	*ALDH* genes are essential in the detoxification of reactive aldehydes produced under oxidative stress conditions during drought Phylogenetic and structural analyses have demonstrated both evolutionary conservation and functional divergence within this gene family up‐regulated *ALDH*s represent promising molecular targets for enhancing drought tolerance through genetic engineering or marker‐assisted breeding strategies This study constitutes the first comprehensive examination of the *ALDH* superfamily in cassava, with significant functional implications for abiotic stress responses	Targets for engineering oxidative stress resilience: The top up‐regulated *ALDH* genes (e.g., *MeALDH7B4*) are excellent candidates for gene editing or transgenic approaches to improve cellular protection under drought	Tran et al. (2025)
RNA‐seq	Under real drought stress: ○ Up‐regulated *YABBY* genes: *MeYABBY02* (+21.38‐fold), *MeYABBY07* (≥ 1.5‐fold), *MeYABBY12* (+7.14‐fold) ○ Down‐regulated *YABBY* genes: *MeYABBY01*, *MeYABBY03* (−43.96‐fold), *MeYABBY04*, *MeYABBY08*, *MeYABBY11* (−35.49‐fold), *MeYABBY13* (−262.66‐fold) ○ Non‐responsive genes: *MeYABBY05*, *MeYABBY06*, *MeYABBY09*, *MeYABBY10* Under PEG6000‐simulated drought (Tissue and time‐dependent responses): ○ *MeYABBY09* (+2.09‐fold) and *MeYABBY10* (+1.84‐fold) were induced in folded leaves ○ Most other *YABBY* genes were down‐regulated in mature leaves and roots ○ *MeYABBY06* displayed dynamic regulation: it was down‐regulated at 3 h but induced at 24 h in folded leaves	The significant up‐regulation of *MeYABBY02* and *MeYABBY12* indicates a potential role in activating protective mechanisms during drought stress, likely enhancing cellular homeostasis or water‐use efficiency The substantial down‐regulation of *MeYABBY13* (−262.66‐fold) and other *YABBY*s may represent a growth‐repression strategy aimed at conserving energy under stress Tissue‐specific expression patterns observed under PEG6000 suggest that *YABBY* transcription factors may regulate stress adaptation differently in developing versus mature tissues Promoter analysis corroborates these findings: *MYBRS* (drought‐responsive) elements were identified in *MeYABBY03* and *MeYABBY06*, whereas *ABRE* (ABA‐responsive) elements were detected in *MeYABBY05*, *MeYABBY06*, and *MeYABBY12*, establishing a link between *YABBY* regulation and ABA‐mediated drought signaling Overall, *YABBY* transcription factors appear to serve dual roles, balancing developmental processes with stress adaptation, in cassava's response to drought	Novel transcription factors for balancing growth and defense: *MeYABBY02/12* are potential targets for MAS to promote stress protection, whereas *MeYABBY13* could be a target for modifying growth‐stress trade‐offs	Van Hai et al. (2025)
RNA‐seq	A total of 77 *bZIP* transcription factor genes have been identified in the cassava genome and categorized into 11 subfamilies Notable *bZIP*s (*MebZIP67*, *MeABF2*, *MeHY5*) exhibit significant up‐regulation in response to drought conditions (induced by mannitol) and ABA treatment. *MebZIP67* was demonstrated to interact with *MeSnRK2.1* physically	*bZIP* transcription factors (TFs) play pivotal roles in ABA‐dependent signaling pathways during drought conditions Promoter analysis demonstrated an enrichment of ABA‐responsive element (*ABRE*), dehydration‐responsive element (*DRE*), and *MYB/MYC* motifs This research provides a foundational basis for the functional characterization of *bZIPs* in the context of cassava improvement	Central regulators for marker development: *MebZIP67* and *MeABF2* are master regulators whose favorable alleles can be selected to enhance the entire ABA signaling pathway in new cultivars	Zeng et al. (2025)
RT‐qPCR	*MeXTH23*, *MeXTH26*, and *MeXTH35* exhibited sustained up‐regulation throughout 24 h *MeXTH5*, *MeXTH11*, *MeXTH12*, *MeXTH28*, *MeXTH29*, and *MeXTH34* showed peak expression at 4 h, followed by a decline Both *MeXTH23* and *MeXTH26* were also activated under salt stress, indicating a shared osmotic stress response	*XTH* enzymes play a role in remodeling the cell wall by modifying xyloglucan networks; their induction under PEG suggests they contribute to maintaining cell wall flexibility and minimizing water loss during dehydration The early transient response (at 4 h) likely reflects initial stress perception, whereas the sustained induction (at 24 h) indicates a role in long‐term acclimation Promoter analysis identified *ABRE*, *ARE*, and *LTR* elements within these genes, linking their expression to ABA signaling and drought‐responsive pathways	Targets for improving water retention: Selecting for sustained expression of *MeXTH23/26/35* could lead to cultivars with more flexible cell walls, better water retention, and reduced wilting	Zhang et al. (2025)

### 2.3 Proteomics

Cassava uses its intricate antioxidant system to respond to drought on several levels (Yan et al. 2021). Proteomic analysis is one of the effective methods for uncovering the function of genes linked to a specific protein in drought responses and adaptation processes (Aslam et al. 2017). Technologies such as two‐dimensional gel electrophoresis (2‐DE) and isobaric tags for relative and absolute quantitation (iTRAQ) have been used as reliable and efficient methods for proteomic analysis; however, they have their limitations. These technologies have also been used in identifying proteins in cassava in response to abiotic stress such as drought, among others (Zhao et al. 2015; Chang et al. 2019; Ding et al. 2019; Yajie et al. 2021). Drought‐responsive proteins involved in the production of secondary metabolism, hormones, heat shock proteins, antioxidant systems, photosynthesis, carbon and nitrogen metabolism, and amino acid metabolism were identified (Table 3). For example, Zhao et al. (2015) profiled proteins in the cassava cultivars ARG7 and SC124 using iTRAQ technology. A total of 561 up‐regulated and down‐regulated proteins displayed significant changes in stressed leaves of ARG7 and/or SC124. Likewise, 150 proteins significantly changed in stressed roots in the same genotypes (Zhao et al. 2015). Chang et al. (2019) used iTRAQ technology to study how cassava SC8 leaves responded to drought stress. Twenty‐six (26) distinct chloroplast proteins were down‐regulated, affecting proteins involved in photosynthesis, which changed the functions of the photosynthetic process.

Besides iTRAQ technology, the use of 2‐DE technology has also been used for proteomics analysis. For instance, Yajie et al. (2021) used 2‐DE technology to assess the drought tolerance of the sexual cassava cultivar versus the SC5 cultivar. They found 34 differently expressed proteins of the glutamine synthetase 2 (*GS2*), regulators of complement (*RCA*), ribulose biphosphate carboxylase small subunit 3B (*RBCS3B*), photosystem II subunit P‐1 (*PSBP‐1*), photosystem II subunit P‐2 (*PSBO2*), triosephosphate isomerase (*TPI*), carbonic anhydrase (*CA1*), and ribulose bisphosphate carboxylase large subunit (*RBCL*) families, which were involved in photosynthesis, ion transport, metabolism, and antioxidants. Notably, proteins involved in photosynthesis were up‐regulated in response to drought stress in the sexual cassava cultivar, indicating that it had a higher ability to sustain normal photosynthetic function under drought stress than SC5. Only a few studies have used 2‐DE to investigate how cassava proteins respond to drought stress. This emphasizes the need for more research to identify additional proteins and their roles, particularly concerning genes involved in protection against or reducing drought stress. These studies showed the efficiency of iTRAQ and 2‐DE technologies in protein analysis, even with their limitations.

New high throughput technologies, such as mass spectrometry, have been developed that enable the profiling of differentially expressed proteins exposed to different environmental stimuli in cassava. These technologies involve the use of liquid chromatography‐mass spectrometry to profile, identify, and understand pathways involved in plants' responses to environmental conditions. For example, a study by Shan et al. (2018) profiled 3339 proteins with peptide sequence coverage varying from 20% to 60% in a Xinxuan 048 (XX048) cassava cultivar. The mentioned proteins play a role in carbohydrate energy metabolism, protein homeostasis, transcription, cell structure, cell membrane transport, signal transduction, stress, and defense responses. Ding et al. (2019) investigated the effects of drought stress on KU50 cassava leaves and roots, and up‐regulated proteins regulated processes like glycolysis, photosynthesis, redox reactions, and reactions to abiotic stress. On the other hand, down‐regulated proteins regulated the secondary metabolism of phenylpropanoids, cell vesicle movement, and protein targeting. Additionally, a study by Guo et al. (2022) reported that proteins that interacted with *CC*‐type *MeGRXC3* affected the catalase activity and drought tolerance in a transgenic cassava cv.60444. Their results showed that under drought stress, *MeGRXC3* negatively regulated the expression of the transcription factor *MeMYB63. MeGRXC3's* interaction with MeTGA2 appears to be the mechanism causing this negative regulation, which in turn causes the down‐regulation of *MeMYB63* expression.

Notably, some of these studies (Zhao et al. 2015; Shan et al. 2018) were among the earliest to use high‐throughput proteomics in the examination of cassava under drought stress, whereas others represent advanced tissue‐specific follow‐up studies. Consequently, the entries in Table 3 illustrate a progress of methodological and biological insights rather than serving solely as a record of initial technological applications. Collectively, these proteomic studies have identified critical proteins associated with photosynthesis, carbon and nitrogen metabolism, hormone signaling, antioxidant systems, and stress defense, which are all elements that are essential to the drought response mechanisms of cassava. To date, only a few studies have reported the use of proteomics approaches to understand cassava response to drought stress, especially in Africa.

**TABLE 3:** Proteomic approaches used to understand cassava response to drought stress.

Proteomic approach	Findings	Description of findings	Implications for breeding	References
iTRAQ and LC–MS	Over 5000 proteins were either up‐regulated or down‐regulated during drought stress	The aquaporin, myoinositol 1‐phosphate synthases, and many proteins involved in the antioxidant systems and secondary metabolism were found Drought‐responsive proteins included protein kinases, two 14–3‐3 proteins, numerous RNA binding proteins and transcription factors, two histone deacetylases, and proteins involved in signaling or gene regulation	System‐wide drought response map: Protein atlas identifies biomarker proteins and key regulators (e.g., 14–3‐3) for phenotyping and manipulation	Zhao et al. (2015)
LC–MS/MS	3339 proteins were discovered, Drought stress changed an abundance of 262 and 296 proteins, respectively, when compared to the control	The proteins play a role in carbohydrate energy metabolism, protein homeostasis, transcription, cell structure, cell membrane transport, signal transduction, stress, and defense responses	Core cellular resilience markers: Focus on genotypes that maintain protein homeostasis and energy metabolism under drought stress	Shan et al. (2018)
iTRAQ and LC–MS/MS	138 up‐regulated and 99 down‐regulated proteins (DEPs) were identified in cassava leaves under drought stress 162 up‐regulated and 145 down‐regulated DEPs were found in the roots of cassava under drought stress	Leaves up‐regulated proteins were significant for glycolysis, photosynthesis, including the Calvin cycle, light reaction, and photorespiration, redox, and abiotic stress Leaves down‐regulated proteins were related to protein targeting, redox, and isoprenoid secondary metabolism Roots up‐regulated proteins were significant for amino acids metabolism, secondary metabolism of flavonoids, signaling of 14–3‐3 proteins, abiotic stress, and TCA transformation; Root down‐regulated proteins were associated with cell vesicle transport, protein targeting, and secondary metabolism of phenylpropanoids	Tissue‐specific breeding targets: Select for root traits (flavonoid metabolism) and leaf traits (photorespiration); 14–3‐3 proteins are central hubs	Ding et al. (2019)
iTRAQ	26 unique chloroplast proteins were identified that respond to drought stress	The drought‐responsive proteins are mostly responsible for photosynthesis, carbon and nitrogen metabolism, and amino acid metabolism Most photosynthesis‐related proteins are down‐regulated with decreases in photosynthetic parameters upon drought stress Numerous proteins associated with carbon and nitrogen metabolism, including rubisco and carbonic anhydrase, were up‐regulated, which may promote cassava drought tolerance by enhancing the carbohydrate conversion efficiency and protecting the plant from oxidative stress	Photosynthetic efficiency targets: Up‐regulation of carbon/nitrogen metabolism proteins (e.g., Rubisco activase) supports higher photosynthetic rates under drought	Chang et al. (2019)
2‐DE	18 proteins were up‐regulated, and 16 proteins were down‐regulated 29 proteins were differentially expressed such as Ribulose bisphosphate carboxylase/oxygenase and photosystem II oxygen‐evolving enhancer protein 1 were up‐regulated in response to drought stress in tetraploid cassava plants	The primary functions of these proteins include photosynthesis, inorganic ion transport and metabolism, carbohydrate synthesis and metabolism, binding, detoxification, and antioxidants	Protective protein markers in tetraploids: Rubisco and PSII enhancer up‐regulation offers genotype‐specific markers for photosynthetic protection	Yajie et al. (2021)
LC–MS/MS	In the leaves during drought, *MeGRXC3* reduced the expression of *MeMYB63* by interacting with *MeTGA2* This interaction suppresses *MeMYB63*, suggesting the presence of a complex drought response network in cassava	Proteins *MeGRXC3* and *MeTGA2* regulate catalase gene expression and transcription factors like MeMYB63 during drought stress in cassava *MeGRXC3* decreases ABA‐induced H_2_O_2_ accumulation in guard cells, affecting stomatal closure, and interacts with catalases *MeCAT1* and *MeCAT2* to regulate their activity. *MeTGA2* interacts with *MeGRXC3* and down‐regulates the expression of *MeMYB63* These proteins are crucial components of the regulatory network controlling drought tolerance and ROS signaling in cassava	Stomatal and Reactive Oxygen Species (ROS) regulation module: *MeGRXC3/MeTGA2* complex is a precise gene editing target to improve water‐use efficiency and antioxidant defense	Guo et al. (2022)

### 2.4 Metabolomics

It is very clear that there is extensive application of metabolomics approaches in the improvement of tropical crops (Ma et al. 2016; Sprenger et al. 2018; Asakura et al. 2021; Zhang et al. 2021; Ghorbanzadeh et al. 2023; Shu et al. 2024). These approaches are particularly effective at identifying, analyzing, and quantifying metabolites and their associated metabolic pathways (Munjal et al. 2022). A crop's entire set of metabolites can be examined by metabolomic approaches, which helps to comprehend metabolomic pathways and pinpoint chemicals that are associated with crop performance and stress tolerance (Ramalingam et al. 2015; Mathur et al. 2021). The approaches rely on three essential technologies: nuclear magnetic resonance spectroscopy, mass spectrometry, and gas chromatographic procedures (Barding et al. 2012).

In cassava, few studies have been reported on the use of metabolomics approaches, especially in response to abiotic stress. For example, Drapal et al. (2018) used Liquid Chromatography‐Mass Spectrometry (LC–MS), Gas Chromatography–Mass Spectrometry (GC–MS), and Ultra‐Performance Liquid Chromatography‐Diode Array Detection (UPLC‐DAD) technologies to classify cassava accessions, including three African accessions: TME3, TMS30555, and TMS60444, grown both in vitro and in the field. The study revealed significant differences in over 100 metabolites, including amino acids, sugars, organic acids, phenylpropanoids, and pigments between field‐grown cassava plant leaf, stem, and root tissues using targeted analysis. Notably, unique metabolic profiles were linked to individual tissues, with main metabolites sharing similarities with in vitro plantlets and field leaf material (Drapal et al. 2018). In addition, South American cassava accession resistant to whitefly infestation was characterized using metabolomics approaches in comparison with the geographically related susceptible accession (Perez‐Fons et al. 2019). Metabolites such as flavonoids and hexose derivatives quercetin occurred in both accessions; however, they were more abundant in the resistant accession, suggesting a defense response. More studies were reported in cassava response to insect feeding, postharvest deterioration, starch quality, and the contributors of provitamin A (Uarrota et al. 2014; Uarrota and Maraschin 2015; Perez‐Fons et al. 2019; Rosado‐Souza et al. 2019; Olayide et al. 2023) using metabolomics approaches; however, few to no reports are available on metabolomic studies in response to drought stress.

In our recent study Mantewu et al. (2025), we focused on the characterization of cassava's metabolic diversity under controlled conditions using an LC–MS‐based untargeted metabolomics approach. We profiled four African genotypes (MSAF1, P4/10, UKF4, and UKF9), detecting over 3300 metabolite features. Major metabolite classes included lipids, phenolics, flavonoids, amino acids, and polyphenolic compounds. Pathway analysis showed significant enrichment in phenylpropanoid biosynthesis, fatty acid degradation, and flavonoid biosynthesis, which are pathways integral to stress adaptation, energy regulation, and antioxidative defense. Notably, the drought‐tolerant genotype P4/10 exhibited higher concentrations of lipid‐ and flavonoid‐associated metabolites. In contrast, UKF4 and UKF9 showed elevated levels of phenolics and nucleotide derivatives related to energy metabolism. These genotype‐specific metabolic profiles demonstrated the capability of metabolomics to elucidate biochemical traits linked to resilience, nutritional quality, and industrial potential in cassava.

These findings become increasingly relevant when contextualized with drought metabolomics from other root and tuber crops and drought‐adapted cereals cultivated in similar African agroecologies. In sweet potato, drought stress induces the accumulation of raffinose‐family oligosaccharides (RFOs), proline, and phenolic acids, which collectively enhance osmotic adjustment and ROS scavenging (Zhou et al. 2022). Under water stress, sorghum maintains high levels of trehalose, polyamines, and hydroxycinnamic acid derivatives, which support cellular homeostasis, root architecture, and photosynthetic efficiency (Behera et al. 2022). The overlap in stress‐associated metabolites, particularly phenylpropanoid‐derived compounds and redox‐active molecules between sorghum and sweet potato, reinforces the hypothesis that flavonoid and phenolic metabolism is a central node in drought adaptation across diverse African crops, whether cereals or tubers.

Collectively, these studies suggest concrete metabolic targets for cassava drought research. Given cassava's high starch turnover and sensitivity to ROS during prolonged drought stress, profiling osmolytes, redox metabolites, and protective secondary metabolites could unveil conserved or unique drought‐adaptive mechanisms. Moreover, integrating tissue‐specific sampling, particularly of roots, which are central to cassava's drought survival strategy, with time‐series metabolomics during progressive drought would reflect successful designs previously established in other crops. Such an approach, applied to various African cassava landraces and improved varieties, including stress‐tolerant lines like P4/10 could identify metabolic signatures predictive of field‐level drought resilience. The foundational metabolic work established by Drapal et al. (2018) and the genotype‐specific metabolic blueprints from Mantewu et al. (2025) together provide an ideal reference framework for such targeted investigations, enabling comparative analyses that link metabolic shifts to agronomic performance under drought conditions in African farming systems.

## 3 Integrating Omics Knowledge With Genome Editing Technologies

**FIGURE 1:**
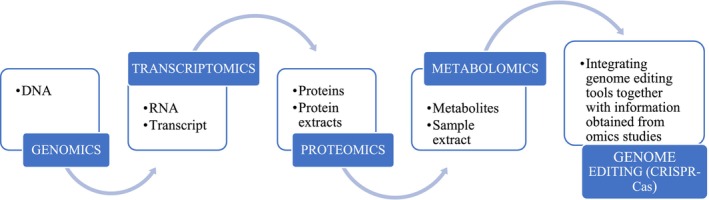
An illustrative depiction outlining the various omics approaches used in crops, leading to the integration of omics insights with genome editing techniques.

To achieve enhanced food security in Africa and possibly realize the United Nations SDG 2 of zero hunger, it is imperative to improve stress resilience to increase crop yields (Bjornlund et al. 2022). An integration of omics and genome editing technologies (Figure 1) can enable the development of varieties that exhibit high productivity and tolerance to environmental conditions (Mmbando et al. 2021). This integration is especially important for enhancing drought tolerance, as omics data can directly inform gene editing initiatives. A great deal of work has been done to characterize cassava germplasms to determine varieties to be included in breeding programs. National Research Agencies in African countries like Uganda (National Crop Resources Research Institute), Tanzania (Tanzania Agriculture Research Institute), Mozambique (International Institute of Tropical Agriculture), South Africa (Agricultural Research Council), and Nigeria (International Institute of Tropical Agriculture) have access to an extensive collection of these materials. By utilizing gene editing tools, it is possible to develop crops capable of tolerating drought stress without compromising their yield or quality.

Genome editing offers significant potential for precisely enhancing traits in cassava, but its effective application requires integration with existing and evolving breeding systems (Fu et al. 2024). Conventional breeding, whereas time‐consuming because of cassava's lengthy breeding cycles and genetic complexity, remains vital, particularly for traits controlled by multiple genes (Abah et al. 2022). Techniques such as MAS and genomic selection (GS) have already accelerated the incorporation of traits like resistance to cassava mosaic disease in African breeding programs (Rabbi et al. 2014; Wolfe et al. 2016). Instead of replacing these methods, genome editing acts as a complementary tool, allowing for the precise introduction or refinement of key genetic variants identified through MAS, GS, or omics approaches (Buch et al. 2023; Yunus et al. 2025). This approach helps avoid issues like linkage drag and enhances elite varieties that are difficult to improve through conventional crosses. Additionally, the clonal propagation of cassava provides a unique advantage, enabling edited elite varieties to be stabilized and distributed without the risk of trait segregation, unlike crops that depend on sexual reproduction (Ceballos et al. 2015).

Genome editing tools have been widely employed to modify targeted genes and their related molecular mechanisms to address environmental stress in plants, which are necessary for the advancement of crop improvement initiatives (Joshi et al. 2020). Genome editing at target sites by specific nucleases has shown great promise for the improvement of crop plants to meet the growing global demands for food. This technology offers an opportunity for biotechnologists to develop a sustainable and prolific agricultural system in terms of improving yield, biotic and abiotic stress tolerance, enhancing resistance to diseases and pests, and modifying plants for product quality. However, the technologies have not been widely explored in tropical crops such as cassava. A recent review has shown that genomic tools have helped to narrow the genomic location of important loci, such as those that confer resistance to major biotic stress, and Cassava mosaic disease (CMD) (Lyons et al. 2021). Furthermore, gene editing procedures that disabled initiation factor 4E (*elF4E*) isoforms, novel cap‐binding protein‐1 (*nCBP‐1*), and novel cap‐binding protein‐2 (*nCBP‐2*) in edited cassava showed a conferred significant viral resistance to Cassava brown streak virus disease (CBSD) (Gomez et al. 2019; Lyons et al. 2021; Mbanjo et al. 2021). The same technology deployed by Gomez et al. (2019) was later used by scientists to develop resistance against a geminivirus, African cassava mosaic virus (ACMV), although the edited cassava plants did not show significant resistance against ACMV (Mehta, Stürchler, et al. 2019). With respect to abiotic stress such as drought, no reports have been recorded. This review highlights the need to integrate these technologies in drought stress breeding programs, especially in Africa. The above information serves as a foundation for possibly conferring drought tolerance in cassava using genome editing tools, together with information obtained from omics studies.

Despite significant advancements in omics‐based characterization of cassava, its application in genome editing for drought tolerance remains limited. Integrating multi‐omics evidence, especially transcriptomic and metabolomic datasets, presents an opportunity to prioritize candidate genes involved in drought‐responsive networks. For instance, genes related to abscisic acid signaling, osmotic adjustment, and ROS detoxification (*LEA*, *DREB*, and *NCED*) consistently appear across omics layers (Ali et al. 2020; Wu et al. 2022). Additionally, metabolite markers such as proline, raffinose, and phenolic derivatives reinforce their functional relevance (Zhou et al. 2022). This cross‐validation supports informed target selection for Clustered Regularly Interspaced Short Palindromic Repeats‐associated (Cas) proteins (CRISPR/Cas) and functional validation, effectively linking omics‐derived genotypic data with observable phenotypic outcomes under drought stress.

Figure 1 illustrates a conceptual framework that connects omics‐driven discovery to genome editing. This framework outlines the progression from identifying candidate genes through integrative analyses such as GWAS, co‐expression, or pathway mapping leading to functional testing and precise modification. Similar workflows have effectively improved drought resilience in crops like rice (*OsNAC14*) and tomato (*SlDREB2*) without sacrificing yield (Shim et al. 2018; Lv et al. 2025), suggesting that a comparable approach could be technically feasible for cassava. However, the successful integration of multi‐omics and genome editing pipelines necessitates a strong bioinformatics infrastructure, reproducibility in data interpretation, and connections to field‐level validation. Additionally, broader considerations such as biosafety evaluation, regulatory compliance, and public acceptance are crucial. These aspects have already been demonstrated by the regulatory approval of gene‐edited crops like the non‐browning mushroom and high‐oleic soybean (Metje‐Sprink et al. 2019), and they will be equally vital for the responsible deployment of edited cassava cultivars within African breeding systems.

## 4 Conclusion and Future Recommendations

The use of advanced omics and genome editing technologies inevitably has enormous potential in controlling and managing cassava abiotic stress; thus, overcoming the limitations of conventional resistance breeding, especially in Africa. The application of omics technologies has allowed a better understanding of drought stress in cassava and provided insight into the biology and taxonomy of this stress (Fu et al. 2016; Mo et al. 2018; Shan et al. 2018; Ding et al. 2019; Wu et al. 2019; Suksamran et al. 2020; Yan et al. 2020; dos Santos Silva et al. 2021; Wang et al. 2021; Guo et al. 2022), however, these technologies have not been fully exploited in Africa. The omics technologies have also given an advantage of exploring the systems biology of the sequenced organisms. Furthermore, advanced gene‐editing techniques such as CRISPR/Cas and Transcription activator‐like effector nucleases (TALEN) provide fundamental breakthroughs in plant genetic improvement that can manipulate desired traits more rapidly and precisely than traditional breeding. In recent years, CRISPR/Cas has been applied in a number of crop systems to improve important agronomic traits such as yield, nutritional value, and disease resistance. The prowess and robustness of the technique hinge on the premise of directly manipulating a target gene in elite varieties; for instance, it can be directly modified by genome editing, thus bypassing the mating procedures. Furthermore, if the target gene is determined, it is independent of plant populations with sufficient genetic variation, and only the sequence information of the target gene is required. In addition, genome editing does not introduce changes beyond the target sites, thus avoiding the potential problems of linkage drag. In the drive to improve the effects of climate change on agricultural crops, incorporating multi‐omics research has enormous promise as seen in other crops like maize, wheat, potato and sweet potato, among others. Future recommendations include using available linked datasets from multi‐omics approaches to properly inform genome editing technologies. By identifying critical genes and molecular pathways linked with drought tolerance, precise genome editing can be performed to create superior cassava varieties with resistance to drought stress. This strategy not only accelerates the breeding process but also ensures the creation of sustainable and robust crops, which are critical for food security in a changing environment.

## Funding

This work was supported by the Water Research Commission (C2023/2024‐01262).

## Consent

The authors have nothing to report.

## Conflicts of Interest

The authors declare no conflicts of interest.

## Data Availability

Data sharing not applicable to this article as no datasets were generated or analyzed during the current study.
